# The Association Between Unhealthy Lifestyle Behaviors and the Prevalence of Chronic Kidney Disease (CKD) in Middle-Aged and Older Men

**DOI:** 10.2188/jea.JE20150202

**Published:** 2016-07-05

**Authors:** Ryoma Michishita, Takuro Matsuda, Shotaro Kawakami, Akira Kiyonaga, Hiroaki Tanaka, Natsumi Morito, Yasuki Higaki

**Affiliations:** 1 Department of Health Development, Institute of Industrial Ecological Sciences, University of Occupational and Environmental Health, Kitakyushu, Fukuoka, Japan; 1 産業医科大学 産業生態科学研究所 健康開発科学研究室; 2 The Fukuoka University Institute for Physical Activity, Fukuoka, Japan; 2 福岡大学基盤研究機関 身体活動研究所; 3 Department of Rehabilitation, Fukuoka University Hospital, Fukuoka, Japan; 3 福岡大学病院リハビリテーション部; 4 Laboratory of Exercise Physiology, Faculty of Health and Sports Science, Fukuoka University, Fukuoka, Japan; 4 福岡大学スポーツ科学部 運動生理学研究室; 5 Fukuoka University Health Care Center, Fukuoka, Japan; 5 福岡大学健康管理センター; 6 Department of Cardiology, Fukuoka University School of Medicine, Fukuoka, Japan; 6 福岡大学医学部 心臓・血管内科学講座

**Keywords:** prevalence of CKD, unhealthy lifestyle behaviors, habitual moderate exercise, late-night dinner, bedtime snacking, CKDの有病, 不健康な生活行動, 運動習慣, 夜遅い夕食習慣, 夜食習慣

## Abstract

**Background:**

This cross-sectional study evaluated the association between unhealthy lifestyle behaviors and the prevalence of chronic kidney disease (CKD) in middle-aged and older men.

**Methods:**

The subjects included 445 men without a history of cardiovascular disease, stroke, or dialysis treatment, who were not taking medications. Unhealthy lifestyle behaviors were evaluated using a standardized self-administered questionnaire and were defined as follows: 1) lack of habitual moderate exercise, 2) lack of daily physical activity, 3) slow walking speed, 4) fast eating speed, 5) late-night dinner, 6) bedtime snacking, and 7) skipping breakfast. The participants were divided into four categories, which were classified into quartile distributions based on the number of unhealthy lifestyle behaviors (0–1, 2, 3, and ≥4 unhealthy behaviors).

**Results:**

According to a multivariate analysis, the odds ratio (OR) for CKD (defined as estimated glomerular filtration rate [eGFR] <60 mL/min/1.73 m^2^ and/or proteinuria) was found to be significantly higher in the ≥4 group than in the 0–1 group (OR 4.67; 95% confidence interval [CI], 1.51–14.40). Moreover, subjects’ lack of habitual moderate exercise (OR 3.06; 95% CI, 1.13–8.32) and presence of late-night dinner (OR 2.84; 95% CI, 1.40–5.75) and bedtime snacking behaviors (OR 2.87; 95% CI, 1.27–6.45) were found to be significantly associated with the prevalence of CKD.

**Conclusions:**

These results suggest that an accumulation of unhealthy lifestyle behaviors, especially those related to lack of habitual moderate exercise and presence of late-night dinner and bedtime snacking may be associated with the prevalence of CKD.

## Abstract (ja)

**はじめに：:**

本研究では、中高年男性の生活行動と慢性腎臓病（CKD）有病との関係について横断的に検討した。

**対象と方法：:**

心疾患や脳血管疾患、人工透析の既往がなく、内服治療を行っていない男性445名を対象に自記式質問票を用いて、1. 運動習慣、2. 身体活動、3. 歩く速さ、4. 食べる速さ、5. 夕食の時間、6. 夜食、7. 朝食欠食の計7項目の生活行動について質問を行った。不健康な生活行動の数により、対象者を0-1個、2個、3個、4個以上の4群に分類した。

**結果：:**

生活行動調査のうち、不健康な生活行動が4個以上ある群は1個以下の群に比べてCKD有病のオッズ比が有意に高かった［オッズ比（OR）：4.67、95%信頼区間（CI）：1.51-14.40、p=0.007］。また、運動習慣のない者（OR：3.06、95% CI：1.13-8.32、p=0.028）、就寝2時間以内に夕食をとることが多い者（OR：2.84、95% CI：1.40-5.75、p=0.004）、夜食習慣を持つ者（OR：2.87、95% CI：1.27-6.45、p=0.011）ほど、CKD有病のオッズ比が有意に高かった。

**まとめ：:**

本研究の結果より、不健康な生活行動が多いこと、とくに運動習慣がないこと、夜遅く夕食をとること、夜食の習慣があることと、CKD有病との関連が示唆された。

## INTRODUCTION

The number of patients with end-stage renal disease (ESRD) in Japan is continuously increasing. Chronic kidney disease (CKD) has been associated with the development of ESRD and cardiovascular disease (CVD).^1^^,^^2^ Currently, the large number of ESRD patients is thought to relate to the increasing number of patients with CKD. Many of the risk factors for CKD are reported to be caused by the aging, hypertension, diabetes mellitus, and metabolic syndrome.^3^^–^^5^

In addition to hypertension, diabetes mellitus, and metabolic syndrome, the incidence of CKD is also closely correlated with unhealthy lifestyle behaviors, such as smoking, heavy alcohol intake, obesity, physical inactivity, and unhealthy diet.^6^^–^^11^ We previously performed a cross-sectional study, which demonstrated that the aerobic capacity and physical activity levels in patients with CKD were significantly lower than in patients without CKD.^12^ Thus, lifestyle modifications in the early stages of hypertension, diabetes mellitus, dyslipidemia, and metabolic syndrome are considered necessary to prevent the development of CKD. However, at present, the accumulation of unhealthy lifestyle behaviors on the risk of CKD has not been studied.

The accumulation of unhealthy lifestyle behaviors has been shown to be associated with the incidence of CVD, stroke, metabolic syndrome, type 2 diabetes, hypertension, and dyslipidemia.^13^^–^^17^ Clarification of the influence of the accumulation of unhealthy lifestyle behaviors on the prevalence of CKD may help to demonstrate the importance of lifestyle modifications in CKD prevention. We considered that unhealthy lifestyle behaviors might be shown to predict the incidence of CKD because an accumulation of unhealthy lifestyle behaviors has been shown to be associated with the development of CVD, stroke, and several coronary risk factors.^13^^–^^17^ We focused on the physical activity, exercise, and eating habits as lifestyle behaviors because increases in the daily physical activity and changes in diet are a very important initial step for the prevention of CVD.^18^ This cross-sectional study was designed to evaluate the association between unhealthy lifestyle behaviors and the prevalence of CKD in middle-aged and older men.

## METHODS

### Subjects

A total of 574 middle-aged and older men received their periodic health check at a health care center in Fukuoka University from 2008 to 2013. Subjects with a past history of CVD (*n* = 9), stroke (*n* = 1), and/or dialysis treatment (*n* = 1), or those taking medications (*n* = 115), such as anti-hypertensive drugs, statins or hypoglycemic agents, were excluded from the analysis because we focused on the effects of lifestyle behaviors without the influence of these medications; women were excluded to remove the influence of gender. A total of 445 men (mean [standard deviation {SD}] age, 50.9 [8.0] years; mean [SD] body mass index [BMI], 23.1 [2.6] kg/m^2^; mean [SD] serum creatinine, 0.87 [0.13] mg/dL; and mean [SD] glomerular filtration rate [GFR], estimated using the Japanese GFR inference formula [eGFR], 75.6 [12.1] mL/min/1.73 m^2^),^19^ with no missing information, were eligible for the present study.

All subjects gave informed consent for participation after agreeing with the purpose, methods, and significance of the study. This study conformed to the Declaration of Helsinki, and was approved by the Ethics Committee of Fukuoka University (No. 11-08-01).

### Blood sampling, blood pressure, and anthropometric measurements

Blood samples were collected early in the morning via venipuncture from an antecubital vein after at least 12 hours of fasting. The blood samples were analyzed by Special Reference Laboratories (SRL Inc., Tokyo, Japan). The serum creatinine, high-density lipoprotein cholesterol (HDL-C), and low-density lipoprotein cholesterol (LDL-C) levels were measured using the direct method. The triglyceride levels were measured using the enzyme method. The plasma glucose was measured using an ultraviolet/hexokinase method, and hemoglobin A_1_c (HbA_1_c) was measured using high-performance liquid chromatography. HbA_1_c is presented as the National Glycohemoglobin Standardization Program (NGSP) value, which was calculated using the conversion equation for HbA_1_c derived by the Japan Diabetes Society (JDS): HbA_1_c (NGSP value; %) = 1.02 × JDS value (%) + 0.25%.^20^

The eGFR was calculated using the Japanese GFR inference formula: eGFR (mL/min/1.73 m^2^) = 194 × serum creatinine (mg/dL)^−1.094^ × age (years)^−0.287^.^21^ The GFR is an accurate measure of renal function^22^ and identifies patients with mild renal impairment despite normal or nearly normal creatinine levels. Moreover, the eGFR is a strong predictor of cardiovascular events and is more useful for this purpose than serum creatinine.^23^^,^^24^ Urinalysis to detect proteinuria was performed using a dipstick, and the urine test results were classified as (−), (±), (1+), (2+), and (3+).^25^ In this study, the CKD was defined according to the definition of the Japanese Society of Nephrology (eGFR <60 mL/min/1.73 m^2^, proteinuria positive [1+ or greater], or both).^19^

Blood pressure was measured in the right arm with the subject sitting in a chair, after more than 5 minutes of rest, and was expressed as an average of duplicate measurements. Height and body weight were measured, and BMI was calculated as the ratio of body weight (kg) to height squared (m^2^). Waist circumference was measured at the level of the umbilicus.

### Assessment of lifestyle behaviors

The subjects’ drinking and smoking habits and lifestyle behaviors regarding exercise, physical activity, and diet were selected for the present study based on the standardized self-administered questionnaire of the National Health Promotion Program, which was started in Japan in fiscal year 2008, and which aimed at preventing CVD, stroke, and metabolic syndrome.^26^^,^^27^ Previous studies have noted that the combination of these lifestyle behaviors is related to mortality and incidence/prevalence of CVD, metabolic syndrome, type 2 diabetes, hypertension, and dyslipidemia.^15^^–^^17^ The subjects’ drinking and smoking habits and lifestyle behaviors were determined based on their responses to the following questionnaire items: 1) habitual moderate exercise ≥30 minutes per session ≥2 times per week (yes or no); 2) physical activity equal to walking at least 1 hour per day (yes or no); 3) walking speed, compared with people of the same sex and age-group (fast or slow); 4) eating speed, compared with others (fast or slow); 5) late-night dinner ≥3 times per week (yes or no); 6) bedtime snacking ≥3 times per week (yes or no); and 7) skipping breakfast ≥3 times per week (yes or no). The subjects drinking and smoking habits were assessed by the following questionnaire items (with “yes” or “no” responses): drinking habit and smoking habit. The total number of unhealthy lifestyle behaviors related to physical activity, exercise, and eating habits (ie, lack of habitual moderate exercise, lack of daily physical activity, slow walking speed, fast eating speed, late-night dinner, bedtime snacking and skipping breakfast) was calculated for each subject.

### Statistical analysis

Data were expressed as the means and SDs. The StatView J-5.0 software package (SAS Institute, Cary, NC, USA) was used for all of the statistical analyses. In this study, the subjects’ drinking and smoking habits and lifestyle behaviors regarding exercise, physical activity, and diet were expressed as category variables, and other coronary risk factors, such as biochemical, blood pressure, and anthropometric indices, were expressed as continuous variables. The inter-group comparisons were performed using the Welch’s *t*-test for continuous variables and the chi-square test for categorical variables. The participants were divided into four categories defined by quartile distributions of the number of unhealthy lifestyle behaviors (0–1, 2, 3, and ≥4 unhealthy behaviors). The inter-multiple group relationships were determined using a one-way repeated measures analysis of variance and the Tukey-Kramer method. A multiple logistic regression analysis was performed to determine the associations between the unhealthy lifestyle behaviors and the prevalence of CKD. In this multiple logistic regression analysis, the level of unhealthy lifestyle behaviors (0–1, 2, 3, and ≥4) was a dependent variable and the prevalence of CKD was an independent variable; age, BMI, smoking habit, drinking habit, triglyceride and HbA_1_c levels, and systolic and diastolic blood pressure were entered as adjusted factors, because these factors potentially influence unhealthy lifestyle behaviors and renal function. A probability value <0.05 was considered to be statistically significant.

## RESULTS

Table 1 compares the subjects’ characteristics and the coronary risk factors between the CKD and non-CKD groups. The characteristics of the individuals with and without CKD were as follows: 39 subjects were found to have CKD (mean [SD] age, 55.1 [6.8] years; mean [SD] BMI, 23.9 [2.2] kg/m^2^; mean [SD] serum creatinine, 1.15 [0.15] mg/dL; mean [SD] eGFR, 53.9 [6.2] mL/min/1.73 m^2^, including 5 subjects with proteinuria), while 406 subjects had normal renal function (mean [SD] age, 50.5 [6.8] years; mean [SD] BMI, 23.1 [2.7] kg/m^2^; mean [SD] serum creatinine, 0.84 [0.09] mg/dL; mean [SD] eGFR, 77.7 [10.3] mL/min/1.73 m^2^). Subjects were categorized by grade of CKD^18^ as follows: 45 participants (10.1%) were G1 (eGFR ≥90 mL/min/1.73 m^2^); 361 subjects (81.1%) were G2 (eGFR 60–89 mL/min/1.73 m^2^); 34 subjects (7.6%) were G3a (eGFR 45–59 mL/min/1.73 m^2^); and 5 subjects (1.1%) were G3b (eGFR 30–44 mL/min/1.73 m^2^). The serum creatinine level, age, BMI, systolic blood pressure, diastolic blood pressure, and HbA_1_c level were significantly higher and eGFR was significantly lower in the CKD group than in the non-CKD group (*P* < 0.05).

**Table 1.** Characteristics of subjects in the CKD and non-CKD groups

	CKD group (*n* = 39)	non-CKD group (*n* = 406)	*P* value
eGFR, mL/min/1.73 m^2^	53.9 (6.2)	77.7 (10.3)	<0.0001
Serum creatinine, mg/dL	1.15 (0.15)	0.84 (0.09)	<0.0001
Age, years	55.1 (6.8)	50.5 (6.8)	0.0004
Body weight, kg	69.0 (8.0)	67.2 (9.1)	0.208
BMI, kg/m^2^	23.9 (2.2)	23.1 (2.7)	0.038
Waist circumference, cm	84.8 (5.6)	82.8 (7.5)	0.070
Systolic blood pressure, mm Hg	129.9 (15.3)	123.8 (14.9)	0.008
Diastolic blood pressure, mm Hg	84.8 (11.0)	79.9 (10.9)	0.002
LDL-C, mg/dL	121.5 (32.4)	119.2 (32.3)	0.468
HDL-C, mg/dL	64.0 (27.8)	63.0 (22.5)	0.690
Triglyceride, mg/dL	123.6 (81.1)	109.8 (78.1)	0.323
Fasting glucose, mg/dL	99.7 (11.6)	92.2 (25.2)	0.074
HbA_1_c, NGSP values; %	5.6 (0.3)	5.5 (0.5)	0.022
Smoking habit, yes/no; *n* (%)	7 (17.9)/32 (82.1)	79 (19.5)/327 (80.5)	0.820
Drinking habit, yes/no; *n* (%)	31 (79.5)/8 (20.5)	314 (77.3)/92 (22.7)	0.759

Data are expressed as mean (SD) or number of subjects (%).

BMI, body mass index; CKD, chronic kidney disease; eGFR, estimated glomerular filtration rate; HbA_1_c, hemoglobin A_1_c; HDL-C, high-density lipoprotein cholesterol; LDL-C, low-density lipoprotein cholesterol; NGSP, national glycohemoglobin standardization program; SD, standard deviation.

Table 2 compares lifestyle behaviors between the CKD and non-CKD groups. The unhealthy lifestyle behaviors regarding a lack of habitual moderate exercise (84.6% vs 63.8%, *P* = 0.009), a lack of daily physical activity equal to walking (79.5% vs 60.1%, *P* = 0.017), late-night dinner (56.4% vs 30.0%, *P* = 0.0008), and bedtime snacking (30.8% vs 11.3%, *P* = 0.0006) were significantly more common in the CKD group than in the non-CKD group, while the total number of unhealthy lifestyle behaviors was also significantly higher in the CKD group than in the non-CKD group (*P* = 0.001). However, there were no significant differences in the other lifestyle behaviors, including slow walking speed, fast eating speed, or skipping breakfast, between CKD and non-CKD groups.

**Table 2.** Differences in lifestyle behaviors of subjects in the CKD and non-CKD groups

	CKD group (*n* = 39)	Non-CKD group (*n* = 406)	*P* value
Habitual moderate exercise: ≥30 min/session and ≥2 times/week, yes/no; *n* (%)	6 (15.4)/33 (84.6)	147 (36.2)/259 (63.8)	0.009
Physical activity equal to walking at least 1 h/day, yes/no; *n* (%)	8 (20.5)/31 (79.5)	162 (39.9)/244 (60.1)	0.017
Walking speed compared with people of the same sex and age-group, fast/slow; *n* (%)	27 (69.2)/12 (30.8)	253 (62.3)/153 (37.7)	0.393
Eating speed compared with others, fast/slow; *n* (%)	18 (46.2)/21 (53.8)	145 (35.7)/261 (64.3)	0.196
Late-night dinners ≥3 times/week, yes/no; *n* (%)	22 (56.4)/17 (43.6)	122 (30.0)/284 (70.0)	0.0008
Bedtime snacking ≥3 times/week, yes/no; *n* (%)	12 (30.8)/27 (69.2)	46 (11.3)/360 (88.7)	0.0006
Skipping breakfast ≥3 times/week, yes/no; *n* (%)	3 (7.7)/36 (92.3)	41 (10.1)/365 (89.9)	0.631
Number of unhealthy lifestyle behaviors	3.4 (1.4)	2.5 (1.3)	0.001

Data are expressed as the mean (SD) and or number of subjects (%) who indicated an unhealthy lifestyle behavior for each status.

SD, standard deviation.

Table 3 compares the subjects’ characteristics and coronary risk factors among the four levels of unhealthy lifestyle behaviors. Serum creatinine level, body weight, BMI, waist circumference, and the triglyceride level were higher and eGFR was significantly lower in the ≥4 group than in the 0–1 group (*P* < 0.05). There were no significant differences in the other coronary risk factors among the four groups.

**Table 3.** Differences in characteristics of subjects in the four unhealthy lifestyle behavior groups

	All (*n* = 445)	Number of unhealthy lifestyle behaviors			

		0–1 (*n* = 104)	2 (*n* = 106)	3 (*n* = 127)	≥4 (*n* = 108)
eGFR, mL/min/1.73 m^2^	75.6 (12.1)	77.9 (10.8)	74.0 (11.2)	77.1 (12.3)	73.3 (13.2)^a^
Serum creatinine, mg/dL	0.87 (0.13)	0.83 (0.11)	0.88 (0.13)	0.85 (0.13)	0.90 (0.15)^a^
Age, years	50.9 (8.0)	51.9 (8.6)	50.1 (8.0)	51.0 (7.5)	50.7 (7.9)
Body weight, kg	67.3 (9.0)	66.0 (9.5)	66.2 (8.9)	67.1 (8.5)	70.0 (8.8)^a^
BMI, kg/m^2^	23.1 (2.6)	22.8 (2.7)	22.9 (2.5)	22.9 (2.4)	24.1 (2.8)^a^
Waist circumference, cm	83.0 (7.4)	81.6 (7.2)	82.4 (7.2)	82.6 (7.4)	85.2 (7.2)^a^
Systolic blood pressure, mm Hg	124.4 (15.0)	125.3 (14.5)	124.9 (15.7)	123.4 (14.0)	124.1 (16.0)
Diastolic blood pressure, mm Hg	80.3 (11.0)	79.4 (10.3)	80.0 (11.0)	80.9 (10.9)	80.8 (11.7)
LDL-C, mg/dL	119.4 (32.3)	115.2 (33.0)	118.2 (30.0)	124.3 (35.8)	118.9 (29.0)
HDL-C, mg/dL	63.1 (22.9)	66.9 (22.3)	62.9 (20.0)	63.5 (26.2)	59.2 (21.9)
Triglyceride, mg/dL	111.0 (78.4)	97.0 (67.1)	105.7 (76.2)	108.4 (63.3)	132.5 (100.3)^a^
Fasting glucose, mg/dL	92.8 (24.4)	91.3 (27.5)	90.6 (25.5)	93.9 (25.1)	95.2 (18.7)
HbA_1_c, NGSP values; %	5.1 (0.5)	5.2 (0.7)	5.1 (0.5)	5.1 (0.4)	5.2 (0.4)
Smoking habit, yes/no; *n* (%)	86 (19.3)/359 (80.7)	21 (20.2)/83 (79.8)	20 (18.9)/86 (81.1)	20 (15.7)/107 (84.3)	25 (23.1)/83 (76.9)
Drinking habit, yes/no; *n* (%)	345 (77.5)/100 (22.5)	84 (80.8)/20 (19.2)	84 (79.2)/22 (20.8)	99 (78.0)/28 (22.0)	78 (72.2)/30 (27.8)

Data are expressed as mean (SD) or number of subjects (%).

BMI, body mass index; eGFR, estimated glomerular filtration rate; HbA_1_c, hemoglobin A_1_c; HDL-C, high-density lipoprotein Cholesterol; LDL-C, low-density lipoprotein cholesterol; NGSP, national glycohemoglobin standardization program; SD, standard deviation.

^a^*P* < 0.05, compared to the 0–1 group.

**Figure 1.**
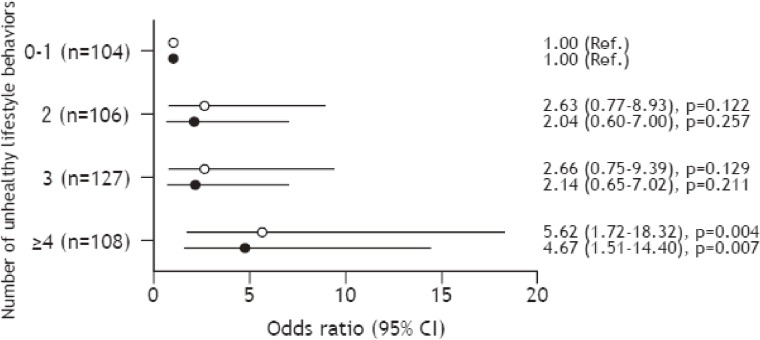
The association between unhealthy lifestyle behaviors and prevalence of CKD. Data are expressed as odds ratio (95% confidence interval [CI]). ○ Univariate model; ● Multivariate model: adjusted for age, body mass index, smoking habit, drinking habit, the triglyceride and HbA_1_c levels, and systolic and diastolic blood pressure.

Figure 1 shows the differences in the odds ratios (ORs) for the prevalence of CKD among the four levels of unhealthy lifestyle behaviors. In a univariate analysis, the OR for CKD was significantly higher in the ≥4 group than in the 0–1 group (OR 5.62; 95% confidence interval [CI], 1.72–18.32). After adjusting for age, BMI, smoking habit, drinking habit, triglyceride, HbA_1_c, and systolic and diastolic blood pressure, the OR for CKD was significantly higher in the ≥4 group than in the 0–1 group (OR 4.67; 95% CI, 1.51–14.40).

**Figure 2.**
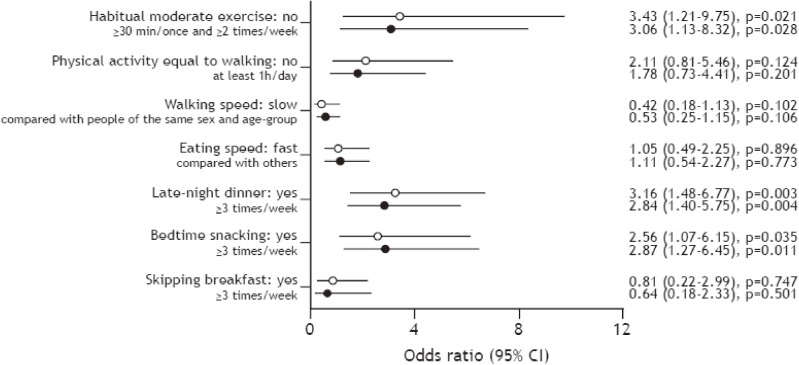
The influence of unhealthy lifestyle behaviors on the prevalence of CKD. Data are expressed as odds ratio (95% confidence interval [CI]). ○ Univariate model; ● Multivariate model: adjusted for age, body mass index, smoking habit, drinking habit, the triglyceride and HbA_1_c levels, and systolic and diastolic blood pressure.

Figure 2 shows the influence of unhealthy lifestyle behaviors on the prevalence of CKD. In a univariate analysis, lack of habitual moderate exercise (OR 3.43; 95% CI, 1.21–9.75), late-night dinner (OR 3.16; 95% CI, 1.48–6.77), and bedtime snacking (OR 2.56; 95% CI, 1.07–6.15) were significantly correlated with the prevalence of CKD. Likewise, after adjusting for age, BMI, smoking habit, drinking habit, triglyceride and HbA_1_c levels, and systolic and diastolic blood pressure, lack of habitual moderate exercise (OR 3.06; 95% CI, 1.13–8.32), late-night dinner (OR 2.84; 95% CI, 1.40–5.75), and bedtime snacking (OR 2.87; 95% CI, 1.27–6.45) were found to be significantly associated with the prevalence of CKD.

## DISCUSSION

The major finding of our study was that the number of unhealthy lifestyle behaviors was significantly higher in the CKD group than in the non-CKD group. Furthermore, after adjusting for the age, BMI, smoking habit, drinking habit, triglyceride and HbA_1_c levels, and systolic and diastolic blood pressure, an increase in the number of unhealthy lifestyle behaviors was found to be associated with an increase in the prevalence of CKD.

It is well known that the accumulation of unhealthy lifestyle behaviors is associated with increased incidence of non-communicable diseases, including metabolic syndrome, type 2 diabetes, hypertension, and dyslipidemia. We can assume that the relationship between unhealthy lifestyle behaviors and CKD has the same mechanisms, and that the accumulation of unhealthy lifestyle behaviors plays a pivotal role in development of these diseases. However, the causality in the relationship of unhealthy lifestyle behaviors with the prevalence of CKD could not be elucidated, due to the cross-sectional design of the present study. In a cross-sectional study using health check-up data, Fujibayashi et al^28^ investigated the association of unhealthy lifestyle behaviors with proteinuria and eGFR and showed that the incidence of proteinuria and a low eGFR (<60 mL/min/1.73 m^2^) increased with increasing number of unhealthy lifestyle factors, and that proteinuria and low eGFR were related to obesity, smoking, eating irregular meals, sleeping less than 5 hours a day, and exercising less than once a week. We found that the relative risk for the prevalence of CKD was significantly elevated by an increase in the number of unhealthy lifestyle behaviors. This finding is consistent with the findings of previous studies. Therefore, the results of the present study were considered to support the possibility that the accumulation of unhealthy lifestyle behaviors is an independent risk factor for the development of CKD. Based on these findings, not only individual lifestyle behaviors but also the accumulation of unhealthy lifestyle behaviors may be associated with the incidence of CKD, ESRD, and CVD.

According to our data, unhealthy lifestyle behaviors related to lack of habitual moderate exercise, lack of daily physical activity, late-night dinner, and bedtime snacking were significantly higher in the CKD group than in the non-CKD group, while in a multivariate analysis, which was adjusted for age, BMI, smoking and drinking habits, triglyceride and HbA_1_c levels, and systolic and diastolic blood pressure, only lack of habitual moderate exercise, late-night dinner, and bedtime snacking were significantly associated with the prevalence of CKD. Notably, there were no significant differences between the CKD and non-CKD groups with regard to the other assessed lifestyle behaviors, such as slow walking speed, fast eating speed, and skipping breakfast. Recently, several studies have demonstrated that individual lifestyle behaviors related to exercise, physical activity, and diet are correlated with the prevalence of CKD. Robinson-Cohen et al^29^ observed that each 60-minute increment in weekly physical activity was associated with a 0.5% decline in eGFR per year. In addition, a previous meta-analysis showed that a lack of habitual exercise and decreased physical activity influence the development of CKD through obesity, hypertension, and type 2 diabetes.^9^ Moreover, it has been reported that an unhealthy diet including high levels of dietary animal fat, sodium, and soft drink consumption leads to the development of renal dysfunction.^30^^–^^32^ Kutsuma et al^33^ showed that, in Japanese adults, the combination of skipping breakfast and late-night dinners had a greater association with the prevalence of metabolic syndrome and proteinuria than skipping breakfast or late-night dinner alone. Based on these findings, the combination of regular habitual exercise and healthy eating habits may be important for preventing the development of CKD.

Previous studies have noted that lack of habitual moderate exercise, late-night dinner, and bedtime snacking were related to mortality and the incidence/prevalence of CVD, metabolic syndrome, type 2 diabetes, hypertension, and dyslipidemia.^34^^–^^36^ The current findings suggest that lack of habitual moderate exercise, late-night dinner, and bedtime snacking may be independent risk factors for the development of CKD, and that they may indirectly influence the development of subsequent ESRD and CVD in middle-aged and older men. Therefore, we consider that the assessment of unhealthy lifestyle behaviors, with a particular focus on regular habitual exercise and healthy eating habits (such as avoiding late-night dinners and bedtime snacking) is necessary when performing lifestyle counseling to prevent the early stages of CKD.

### Study limitations and clinical implications

There are several limitations to the present study. First, the limited study population included a small number of male subjects, who were predominantly middle-aged and older, who were not taking any medications, and who did not have any health complications. Thus, our study contains selection bias, because our limited study population may include more CKD subjects with very slowly declining renal function than CKD subjects with fast progression. Therefore, it remains unclear whether our findings are applicable to women, patients with ESRD, or those who have other complications. Second, due to the cross-sectional design of the study, it was not possible to clarify the causality of the relationship between the unhealthy lifestyle behaviors and the prevalence of CKD. Third, while renal function is influenced by dietary electrolytes, the present study could not confirm the dietary total energy or sodium and potassium intake of the subjects. Therefore, we could not clarify the influence of dietary electrolytes using the subjects’ dietary records. Finally, we assessed the eGFR using the Japanese GFR inference formula^21^ and used proteinuria as an index of renal function. To more fully evaluate the influence of unhealthy lifestyle behaviors on renal function, other indices of renal function, such as urinary protein excretion, microalbuminuria, or cystatin C should be simultaneously assessed. We could not measure these additional markers of renal function because this study was performed within the constraints of the health check-up.

However, this study is the first report to evaluate the association between accumulation of unhealthy lifestyle behaviors and the prevalence of CKD. Serum creatinine level can be easily measured as part of a routine clinical evaluation. However, eGFR is a strong predictor of cardiovascular events and is more useful for this purpose than serum creatinine level.^23^^,^^24^ Furthermore, in several recent studies, it has been clearly demonstrated that the incidence of CKD is also closely correlated with unhealthy lifestyles behaviors, such as smoking, heavy alcohol intake, obesity, physical inactivity, and unhealthy diet.^6^^–^^11^ Therefore, the results of the present study show a link between the accumulation of unhealthy lifestyle behaviors and the prevalence of CKD and may support the hypothesis that the accumulation of unhealthy lifestyle behaviors leads to an increase in incidence of CVD and development of ESRD. Based on our results, we consider it necessary to perform lifestyle counseling, especially counseling that focuses on regular habitual exercise and healthy eating habits (such as avoiding late-night dinners and bedtime snacking), to reduce the incidence of CKD. Additional research in a larger sample is necessary to more precisely clarify the mechanisms, clinical implications, and associations between unhealthy lifestyle behaviors and the incidence of CKD following long-term intervention. In particular, the number of patients with ESRD in Japan is on the rise with the increase in prevalence of type 2 diabetes, so further studies are also needed to clarify the influence of unhealthy lifestyle behaviors on CKD in subjects with type 2 diabetes.

### Conclusions

This study was designed to evaluate the association between unhealthy lifestyle behaviors and the prevalence of CKD in middle-aged and older men. We found that the OR for CKD significantly increased with an increase in the number of unhealthy lifestyle behaviors. Furthermore, after adjusting for age, BMI, smoking, drinking habits, triglyceride and HbA_1_c levels, and systolic and diastolic blood pressure, we found that a lack of habitual moderate exercise, late-night dinner, and bedtime snacking were significantly associated with the prevalence of CKD. These results suggest that an accumulation of unhealthy lifestyle behaviors, especially lack of habitual moderate exercise, late-night dinner, and bedtime snacking, may be associated with the prevalence of CKD.

## ONLINE ONLY MATERIAL

Abstract in Japanese.
