# Supplementary material for: The Association Between Unhealthy Lifestyle Behaviors and the Prevalence of Chronic Kidney Disease (CKD) in Middle-Aged and Older Men
Source: J Epidemiol. 2016 Jul 5;26(7):378–85. doi: 10.2188/jea.JE20150202 (PMC4919483; doi:10.2188/jea.JE20150202)
Supplement: Abstract in Japanese. [file je-26-378-s001.pdf]

中高年男性の不健康な生活行動と慢性腎臓病（CKD）の有病との関係

道下竜馬[1、2]、松田拓朗[2、3]、川上翔太郎[2、4]、清永明[2、4]、田中宏暁[2、4]、
森戸夏美[5、6]、檜垣靖樹[2、4]

1. 産業医科大学 産業生態科学研究所 健康開発科学研究室
2. 福岡大学基盤研究機関 身体活動研究所
3. 福岡大学病院リハビリテーション部
4. 福岡大学スポーツ科学部 運動生理学研究室
5. 福岡大学健康管理センター
6. 福岡大学医学部 心臓・血管内科学講座

はじめに：本研究では、中高年男性の生活行動と慢性腎臓病（CKD）有病との関係について横断的に検討した。

対象と方法：心疾患や脳血管疾患、人工透析の既往がなく、内服治療を行っていない男性445名を対象に自記式質問票を用いて、1. 運動習慣、2. 身体活動、3. 歩く速さ、4. 食べる速さ、5. 夕食の時間、6. 夜食、7. 朝食欠食の計7項目の生活行動について質問を行った。不健康な生活行動の数により、対象者を0-1個、2個、3個、4個以上の4群に分類した。

結果：生活行動調査のうち、不健康な生活行動が4個以上ある群は1個以下の群に比べてCKD有病のオッズ比が有意に高かった［オッズ比（OR）：4.67、95%信頼区間（CI）：1.51-14.40、p=0.007］。また、運動習慣のない者（OR：3.06、95% CI：1.13-8.32、p=0.028）、就寝2時間以内に夕食をとることが多い者（OR：2.84、95% CI：1.40-5.75、p=0.004）、夜食習慣を持つ者（OR：2.87、95% CI：1.27-6.45、p=0.011）ほど、CKD有病のオッズ比が有意に高かった。

まとめ：本研究の結果より、不健康な生活行動が多いこと、とくに運動習慣がないこと、夜遅く夕食をとること、夜食の習慣があることと、CKD有病との関連が示唆された。

キーワード：CKDの有病、不健康な生活行動、運動習慣、夜遅い夕食習慣、夜食習慣
